# Supplementary material for: The Effect of Metal Cations on the Aqueous Behavior of Dopamine. Thermodynamic Investigation of the Binary and Ternary Interactions with Cd2+, Cu2+ and UO22+ in NaCl at Different Ionic Strengths and Temperatures
Source: Molecules. 2021 Dec 19;26(24):7679. doi: 10.3390/molecules26247679 (PMC8709034; doi:10.3390/molecules26247679)
Supplement: Supplementary file 1 [file molecules-26-07679-s001.zip › molecules-1485928-supplementary.pdf]

Table S1. Calculated formation constants[a)] of the Cd^{2+}/Dop^- species in NaCl aqueous solutions in molal concentration scale

**I/mol Kg^{-1}**	**logβ_{ML}**	**logβ_{MLH}**	**logβ_{ML2}**
		T = 288.15 K	
0.150	6.65	14.89	11.61
		T = 298.15 K	
0.149	6.48	14.08	10.88
0.494	6.05	14.17	10.30
0.742	6.40	14.70	10.34
0.976	6.29	14.08	9.11
		T = 310.15 K	
0.150	4.60	12.55	9.25

[a)] Refer to the general equilibrium: p M^{n+} + q L^{z-} + r H^+ = $M_pL_qH_r^{(np-zq+r)}$.

Table S2. Calculated formation constants[a)] of the Cu^{2+}/Dop^{-} species in NaCl aqueous solutions in molal concentration scale

I/mol kg^{-1}	logβ_{ML2}	logβ_{M2L}	logβ_{M2L2}	log$\beta_{M2L2(OH)2}$	log$\beta_{M2L(OH)}$	log$\beta_{ML2(OH)}$
T = 288.15 K						
0.162	21.02	15.21	27.33	13.40	8.87	11.38
0.496	20.49	15.32	26.85	12.67	9.02	11.24
0.753	20.20	15.41	26.59	12.26	9.20	11.21
1.013	19.94	15.50	26.35	11.88	9.41	11.20
T = 298.15 K						
0.172	19.35	14.57	25.66	11.72	8.86	11.01
0.480	18.87	14.68	25.20	11.04	9.00	10.88
0.750	18.55	14.77	24.92	10.60	9.19	10.84
0.985	18.31	14.85	24.71	10.26	9.37	10.83
T = 310.15 K						
0.146	17.58	13.84	23.86	9.94	8.85	10.62
0.490	17.01	13.96	23.33	9.16	8.98	10.46
0.743	16.71	14.05	23.06	8.73	9.15	10.42
1.008	16.44	14.14	22.82	8.35	9.36	10.40
T = 318.15 K						
0.163	16.39	13.40	22.66	8.73	8.84	
0.515	15.81	13.52	22.12	7.93	8.98	10.18
0.750	15.54	13.60	21.88	7.54		
1.024	15.26	13.69	21.62	7.14	9.34	10.13

[a)] Refer to the general equilibrium: $p\ M^{n+} + q\ L^{z-} + r\ H^{+} = M_pL_qH_r^{(np-zq+r)}$.

Table S3. Calculated formation constants of the UO_2^{2+}/Dop^- species in NaCl aqueous solutions at different ionic strengths and temperatures, in molar concentration scale

I/mol kg^{-1}	logβ_{ML2} [a)]	logβ_{MLAc} [a)]	logβ_{MLOH} [a)]
***T* = 288.15 K**			
0.162	21.50	16.10	6.66
0.531	21.87	15.87	6.71
0.752	22.24	15.82	6.83
1.012	22.65	15.80	6.96
***T* = 298.15 K**			
0.166	21.68	16.13	7.09
0.510	21.34	15.65	6.83
0.752	21.21	15.42	6.73
1.013	21.12	15.23	6.64
***T* = 310.15 K**			
0.162	22.14	16.57	7.48
0.513	21.86	16.26	7.29
0.754	21.78	16.15	7.23
1.016	21.72	16.07	7.20
***T* = 318.15 K**			
0.161	22.11	17.21	7.49
0.512	21.96	16.79	7.51
0.757	21.96	16.60	7.60
1.018	21.99	16.45	7.71

[a)] Refer to the general equilibria: p M^{n+} + q L^{z-} + r H^+ = $M_pL_qH_r^{(np-zq+r)}$ and p M^{n+} + q L^{z-} + r H^+ + Ac^- = $M_pL_qH_rAc^{(np-zq+r-1)}$.

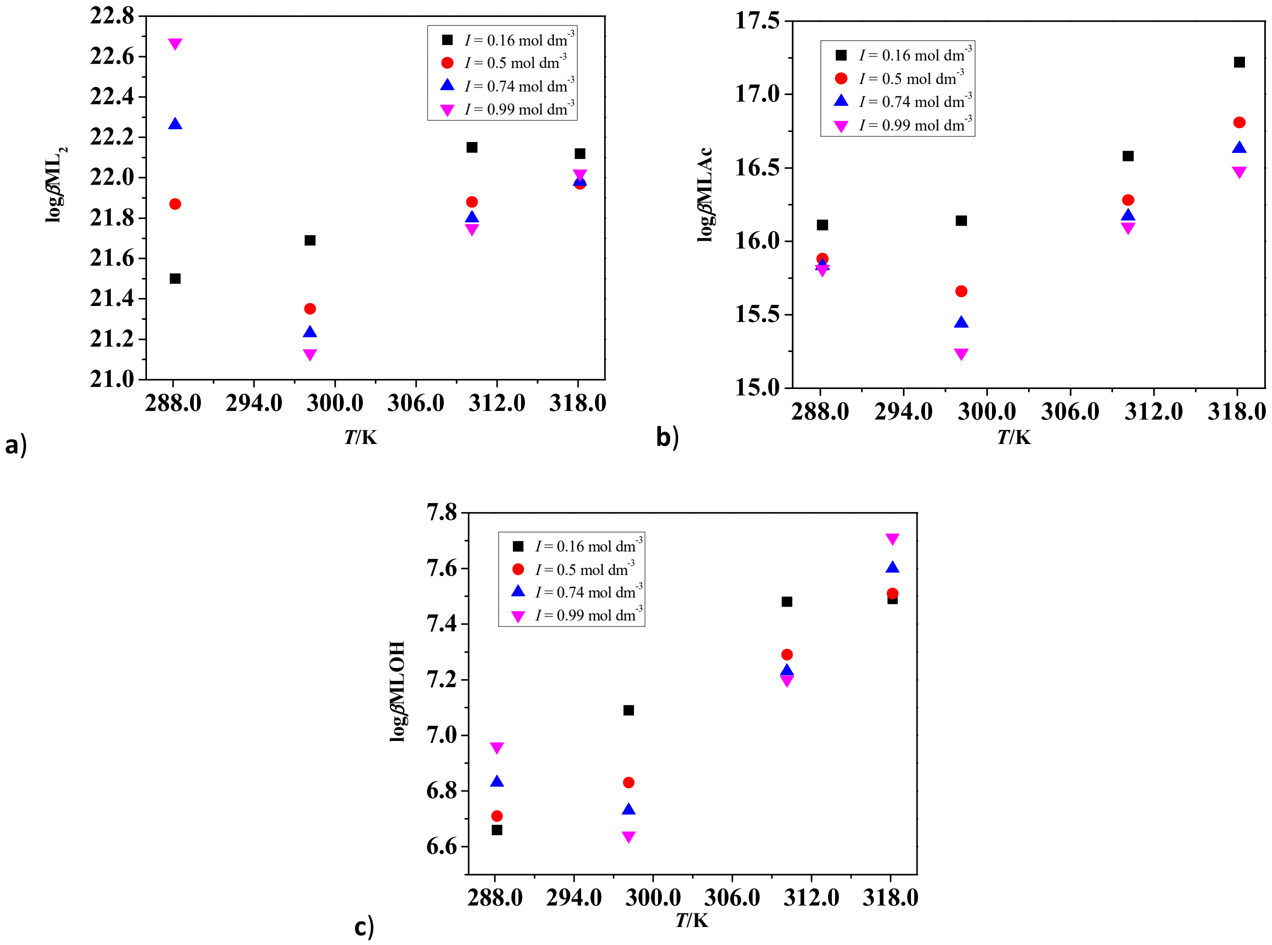

Figure S1. Trend of the experimental formation constants of the UO_2^{2+}/Dop^- complex species, at different temperature and ionic strength values: **a**) ML_2; **b**) MLAc; **c**) MLOH

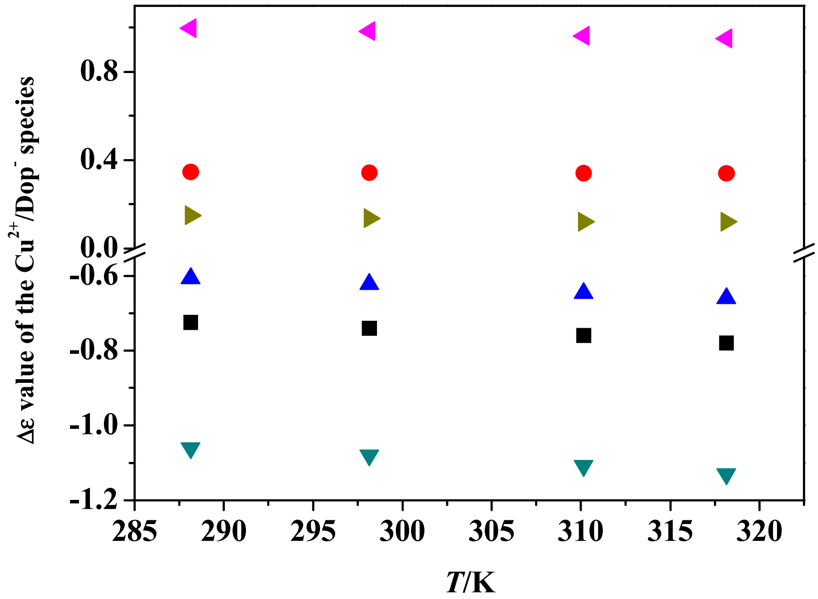

Figure S2. trend of the Δε values of the Cu^{2+}/Dop^{-} species vs *T*/K.

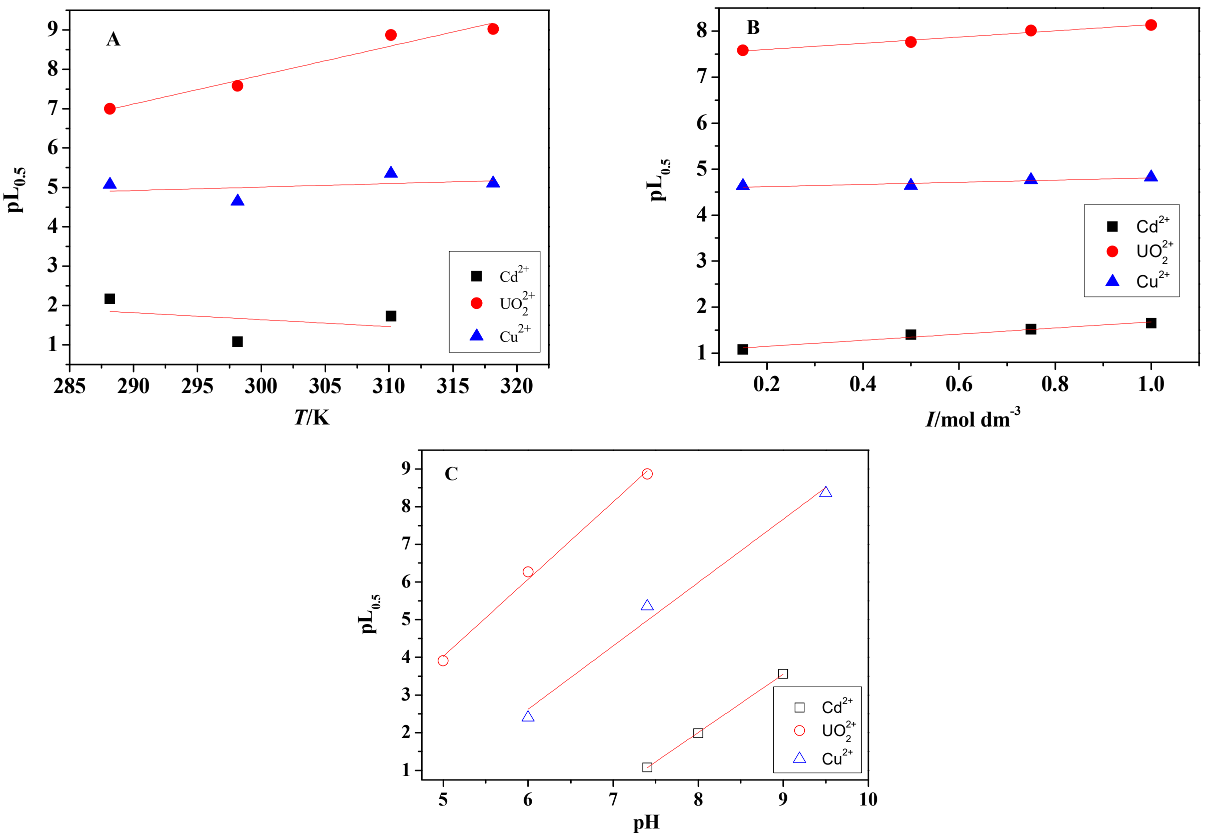

Figure S3. Trend of $pL_{0.5}$ vs **A**) T/K, **B**) I/mol dm^{-3} and **C**) pH.
